# Prevention and treatment of oral mucositis in patients receiving chemotherapy

**DOI:** 10.4317/jced.51313

**Published:** 2014-02-01

**Authors:** Carlos Alvariño-Martín, Maria G. Sarrión-Pérez

**Affiliations:** 1 Dentistry, Master of Oral Medicine and Oral Surgery, Faculty of Medicine and Dentistry, University of Valencia, Spain; 2 Adjunt Professor of the Department of Stomatology, Faculty of Medicine and Dentistry, University of Valencia, Spain

## Abstract

Oral mucositis is one of the most common side effects of cancer treatment (chemotherapy and/or radiotherapy). It is an inflammatory process that affects the mucosa of the oral cavity, giving rise to erythematous areas in combination with ulcers that can reach a large size. The true importance of oral mucositis is the complications it causes – fundamentally intense pain associated to the oral ulcers, and the risk of overinfection. This in turn may require reduction or even suspension of the antineoplastic treatment, with the risk of seriously worsening the patient prognosis. This points to the importance of establishing therapeutic tools of use in the prevention and/or treatment of mucositis. The present study offers a literature review of all the articles published over the last 10 years referred to the prevention and/or treatment of oral mucositis associated to chemotherapy.

** Key words:**Oral mucositis, management, prevention, treatment, chemotherapy.

## Introduction

Oral mucositis (OM) is defined as inflammation of the mucosa oral cavity, and is clinically characterized by the presence of erythematous areas that subsequently merge with ulcerations (1).

Oral mucositis is caused by destruction of the oral mucosal epithelium and suppression of its growth secondary to antineoplastic treatment in the form of chemotherapeutic drug substances or radiotherapy (2,3).

The pathogenesis of mucositis is currently based on a model comprising five biological phases, developed by Sonis et al. (4): initiation, signaling, signal amplification, ulceration and healing.

The frequency of mucositis and its severity are fundamentally dependent upon the type, duration and dose of chemotherapy used (5). In this sense, bone marrow-suppressing (myeloablative) chemotherapy is associated with a mucositis risk of 60-100% (4,6,7), while the combination of chemotherapy and radiotherapy implies a risk of almost 100% (3).

The clinical manifestations of OM become visible 4-5 days after the start of chemotherapy, with the detection of erythematous areas in the oral cavity. After 7-10 days ulcers start to develop; these gradually grow in number and size, and tend to merge, forming large ulcerated zones (4,8). The ulcers are generally of scant depth, with a necrotic base, and the margins show little inflammatory infiltration. These lesions are very painful, cause swallowing problems, and take about two weeks to heal once chemotherapy has been suspended (3,4).

A number of OM classification and staging systems have been described (9), though the most widely used is that proposed by the World Health Organization (WHO)( Table 1).

**Table 1:**
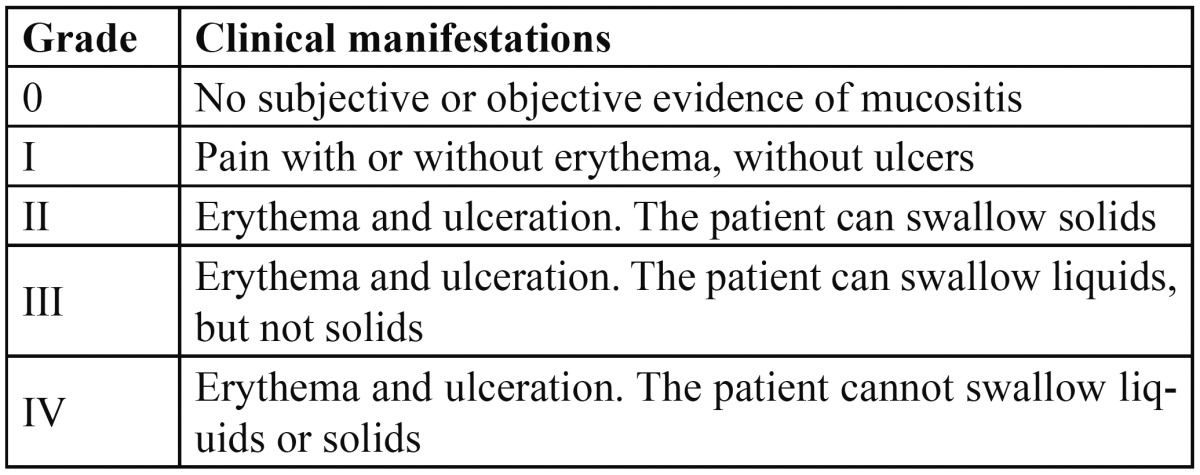
Oral mucositis classification – staging system proposed by the World Health Organization (WHO).

Oral mucositis causes many complications, ranging from speech and swallowing difficulties to intense pain and ulcer over infection, which may lead to systemic infection (bacteremia or fungemia) – posing a threat to patient life and requiring admission to hospital, with the increased economic costs this implies. These complications may require reduction or even suspension of the antineoplastic treatment, with the risk of seriously worsening the patient prognosis (3,5,9,10).

In relation to OM secondary to chemotherapy, many studies have evaluated the efficacy of different interventions designed to prevent and/or treat the disease.

The present review examines those therapeutic tools that have been shown to be useful in the prevention or treatment of OM.

## Material and Methods

A literature search was made of the PubMed, Cochrane and Scopus databases, using different combinations of the following key words: “oral mucositis”, “treatment”, “prevention”, “management”, “chemotherapy”. These key words in turn were validated by the “Mesh” and “DeCS” dictionaries, and the boolean operator “AND” was used to relate them in each search. The limits established for inclusion of the articles in the study were: publications in English, studies in humans, and articles published over the last 10 years [2002-2012].

Following application of the filters, we identified 169 articles. Many of them were discarded, since they only focused on OM associated to radiotherapy. We finally selected only those publications affording a scientific evidence level of I-II according to the classification proposed by the National Health and Medical Research Council of Australia (11), and those articles which while not constituting systematic reviews did specify the material and methods used. A total of 44 articles were thus finally obtained.

## Results

The main strategies and drugs described in the literature over the last 10 years designed to prevent and/or treat OM secondary to chemotherapy are the following:

-Oral hygiene protocols

Oral care can reduce the presence of microbial flora, the pain and bleeding, and prevent infections. Likewise, good oral health reduces the risk of dental complications (6). However, the effectiveness of oral hygiene in preventing the appearance of OM or in reducing its severity has been placed in doubt, since the studies published to date offer contradictory results (12,13).

-Chlorhexidine digluconate

The antimicrobial agent most widely studied in the management of OM is chlorhexidine, used in oral rinses at concentrations of 0.12-0.2%, since it has been suggested to be useful in maintaining improved oral hygiene and in reducing mucosal inflammation (1). However, the results obtained in the different clinical trials are inconclu-sive (1,2,14).

-Cytoprotective agents

•Amifostine:

Amifostine (Ethyol®) is an organic thiophosphate believed to act as a reactive oxygen species (ROS) scavenger. In this context, ROS are known to play a key role in the etiopathogenesis of OM (2,15).

In this review we have found only one randomized, controlled clinical trial (RCCT) published in the last 10 years, which reported no benefits with the preventive use of amifostine in patients subjected to chemotherapy- radiotherapy (16).

•Sucralfate:

Sucralfate is a drug with cytoprotective properties used in the treatment of peptic ulcers (1). Many studies have evaluated its effectiveness in irradiated patients, with very dissimilar results. In contrast, it is little used in pa-tients subjected to chemotherapy. Indeed, over the last decade, only one RCCT has examined the effect of sucralfate rinses in patients subjected to chemotherapy with 5-fluorouracil (5-FU)(17), and its results indicate no effectiveness in the prevention of OM.

•Glutamine:

Glutamine is one of the most abundant amino acids in the body, where it is involved in numerous beneficial functions. It could be of help in the prevention and treatment of OM, since it plays a fundamental role in regulation of the redox potential, and some studies have even suggested that it exerts a favorable effect by reducing the production of proinflammatory cytokines (10).

In the last 10 years three RCCTs (18-20) have evaluated the preventive effects of glutamine in patients scheduled for chemotherapy. Two of them obtained results supporting the use of glutamine, though the samples sizes were relatively small (16-32 subjects)(19,20). In contrast, the study published by Pytlik et al. (18) not only found glutamine to be ineffective in the prevention of OM, but also suggested that it might worsen OM and even increase the risk of tumor relapse.

-Allopurinol

Allopurinol has been used on an experimental basis for both the prevention and treatment of mucositis induced by fluorouracil. Its activity is based on two different mechanisms: the elimination of ROS, and specific inhibition of the activation of fluorouracil.

The clinical trial conducted by Panahi et al. (21) found allopurinol rinses to offer little efficacy in preventing OM in patients subjected to chemotherapy with 5-FU.

-Cryotherapy

The application of ice within the oral cavity causes local vasoconstriction, which in turn lessens blood flow to the oral mucosa and reduces the amount of cytotoxic medication reaching the cells – thereby lowering the incidence of mucositis (22).

This technique involves placing ice cubes in the mouth 5 minutes before starting the chemotherapy cycle, and keeping them in the mouth for 30-45 minutes.

Cryotherapy for the prevention of OM has been studied in application to different chemotherapy regimens ( Table 2). In this context, Sorensen et al. (23), Papadeas et al. (24) and Nikoletti et al. (25) conducted clinical trials in patients treated with 5-FU; Gori et al. (26) studied patients treated with methotrexate; and Lilleby et al. (27) and Svanberg et al. (28) investigated conditioning therapies with melphalan prior to hematopoietic stem cell transplantation (HSCT). The results showed cryotherapy to be effective in the prevention of OM when used with chemotherapeutic agents having a short plasma half-life, such as bolus doses of 5-fluorouracil and melphalan. In contrast, drugs with a half-life of between 3-15 hours, such as methotrexate, yielded inconclusive results.

**Table 2:**
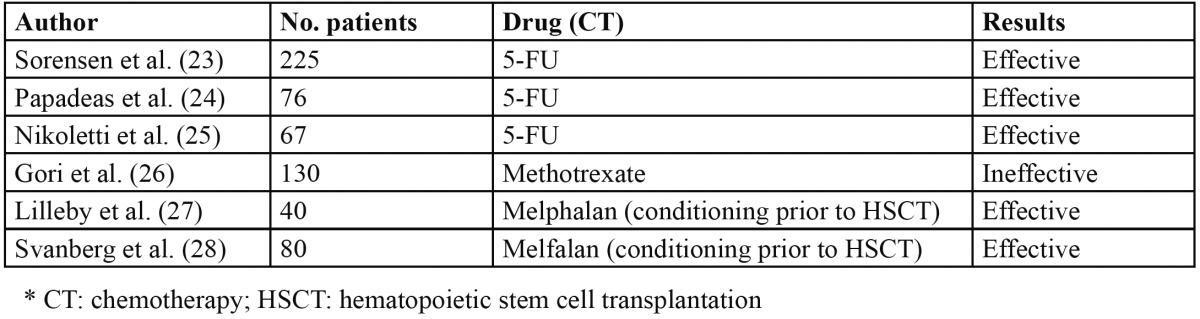
Studies on cryotherapy for the prevention of oral mucositis.

-Growth factors

Growth factors are proteins that stimulate cell growth, proliferation and differentiation. Within this family of proteins, the growth factors most widely investigated in the prevention and treatment of OM are palifermin (keratinocyte growth factor) and the colony-stimulating factors (29).

Palifermin (Kepivance®) is a recombinant human keratinocyte growth factor that stimulates epithelial cell proliferation and increases the thickness of the non-keratinized layers of the oral and gastrointestinal mucosa (29).

In this review we identified 8 RCCTs ( Table 3) in which the efficacy of palifermin in the prevention of OM induced by chemotherapy has been studied (30-37).

**Table 3:**
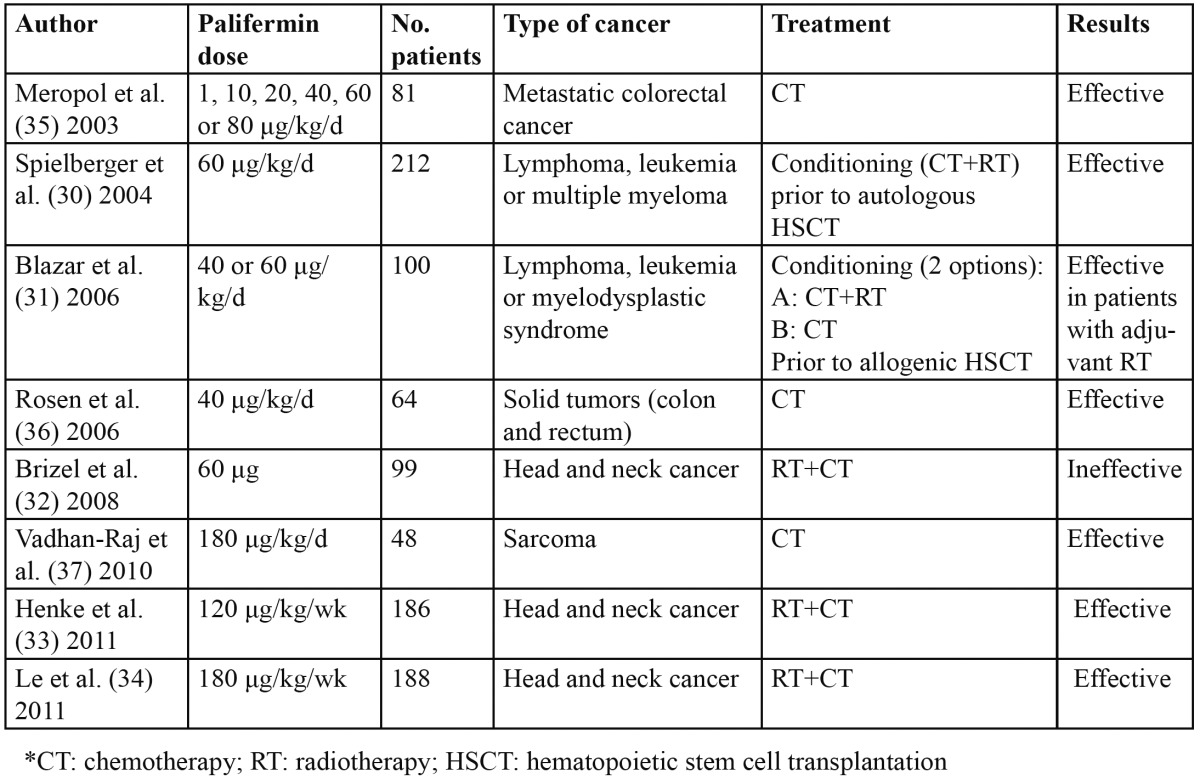
Studies of palifermin in the prevention of oral mucositis.

Although the results have been promising in almost all of these trials, the publication with the strongest met-hodological design was that carried out by Spielberger et al. (30), who found palifermin to be very effective in the prevention of oral mucositis in patients scheduled for conditioning treatment (chemotherapy and total body irradiation) prior to hematopoietic stem cell transplantation.

In contrast, the results obtained are not so good when the patients are subjected to conditioning treatment with chemotherapy but without total body irradiation prior to allogenic stem cell transplantation (31).

Regarding patients with head and neck cancer subjected to chemotherapy – radiotherapy, the results obtained by Brizel et al. (32) reveal no efficacy on the part of palifermin, in contrast to the data obtained by the studies published in 2011 by Henke et al. (33) and Le et al. (34), in which palifermin was seen to significantly reduce the incidence of severe OM. The explanation for these conflicting results may be the possibly insufficient palifermin dose (60 μg in a single dose) administered in the study of Brizel et al.

The studies published by Meropol et al. (35), Rosen et al. (36) and Vadhan-Raj et al. (37) support the efficacy of palifermin in patients with solid tumors (metastatic colorectal cancer or sarcoma) subjected to chemotherapy, though it must be noted that their data may involve bias (masking difficulties, small sample size, etc.).

Colony-stimulating factors are glycoproteins produced by a broad range of human cells, some of which are of hematopoietic lineage, such as fibroblasts, endothelial cells and immune system cells (macrophages, T cells). Interest has focused mainly on granulocyte colony-stimulating factor (G-CSF) and granulocyte-macrophage colony-stimulating factor (GM-CSF). Both act at two levels in the prevention and treatment of mucositis: at central level by stimulating bone marrow recovery, and at peripheral level by promoting keratinocyte production (3,29).

There have been no RCCTs in the last decade on the preventive or therapeutic efficacy of G-CSF in application to mucositis. In relation to GM-CSF, the studies of Dazzi et al. (38) and Valcarcel et al. (39) indicate scant effi-cacy on the part of oral rinses containing GM-CSF in preventing and treating mucositis.

-Low-ievel laser therapy (LLLT)

It is known that radiation, at certain wavelengths, may exert beneficial effects upon cells. Phototherapy, including low-level laser therapy (LLLT), is based on the interaction of low energy density light (a few J/ cm2) with cells and tissues, without the generation of thermal effects. It is believed that this type of therapy, with wavelengths of between 600-900 nm, could exert a biomodulating effect upon cells and tissues secondary to absorption of the light by endogenous photoreceptors (7,40). The activation of these photoreceptors may modify cell metabolism depending on the light energy dose delivered. Accordingly, low energy doses could act by reducing the production of ROS, and also by stimulating protein synthesis with the 

facilitation of tissue repair. Likewise, they may exert an antiinflammatory effect by reducing prostaglandin synthesis (7).

In recent years different studies have been made on the use of LLLT for the prevention and treatment of oral mucositis ( Table 4).

**Table 4:**
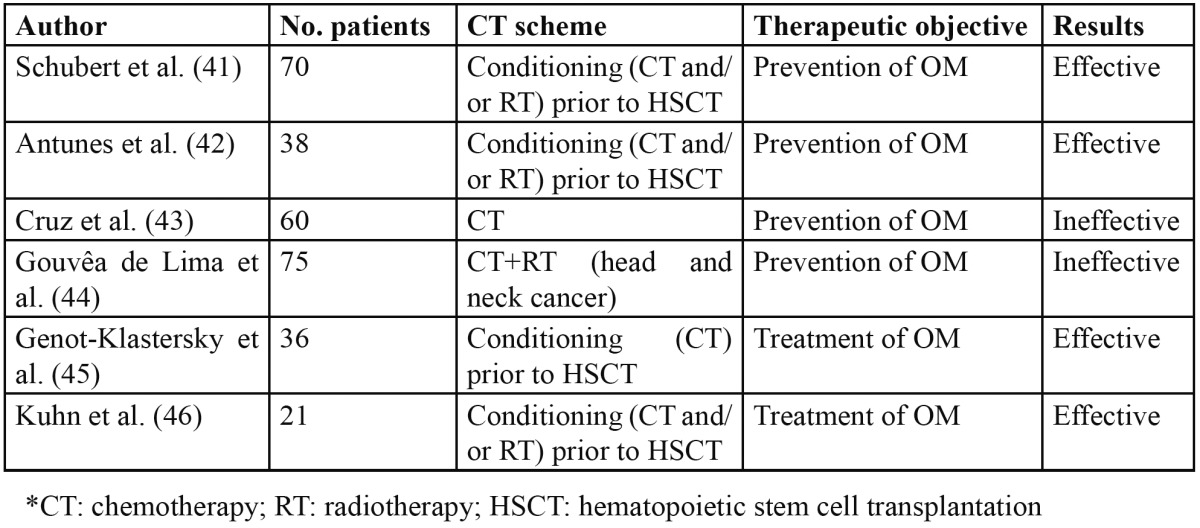
Studies of low-level laser therapy in the prevention and treatment of oral mucositis.

The studies of Schubert et al. (41) and Antunes et al. (42) have demonstrated the efficacy of LLLT at wave-lengths of 650-660 nm in the prevention of OM among patients subjected to conditioning with chemotherapy or chemotherapy - radiotherapy prior to HSCT.

However, the study published by Cruz et al. (43) revealed no benefits with the preventive use of LLLT in pa-tients subjected to different chemotherapy regimens.

The results likewise have not been encouraging in patients with head and neck cancer scheduled for chemotherapy - radiotherapy (44).

In assessing the efficacy of LLLT in the treatment of established mucositis, mention must be made of the studies carried out by Genot-Klastersky et al. (45) and Kuhn et al. (46). Both reported a clear decrease in the duration of OM in the patients treated with LLLT.

## Discussion

On the basis of the data afforded by the reviewed studies, an analysis can be made of their conclusions, comparing them with those of other literature reviews and metaanalyses published over the last 10 years.

Worthington et al. (47) published a systematic review in the Cochrane database referred to randomized studies on the prevention of mucositis, with the inclusion of a total of 131 trials assessing 43 different interventions. Based on all these studies, the authors firstly concluded that only cryotherapy and palifermin offer some evidence of benefit. Secondly, very weak evidence of benefit was observed for 8 interventions (aloe vera, amifostine, glutamine, G-CSF, honey, laser, polymyxin / tobramycin / amphotericin (PTA) paste or tablets, and sucralfate). On the other hand, they pointed out that all 10 interventions were considered to imply a high or uncertain risk of bias.

Regarding the metaanalysis on the treatment of mucositis published by Clarkson et al. (48) in the same database, the authors found that of the 21 interventions included (32 trials), only LLLT showed a certain effectiveness in the treatment of mucositis, based on two studies with no high risk of bias in the results obtained.

It must be underscored that these reviews included interventions aimed at preventing and treating OM induced both by chemotherapy and radiotherapy, while our review focused on the techniques used to prevent or treat mucositis induced only by chemotherapy or by combinations of chemotherapy - radiotherapy. On the other hand, and since there are many chemotherapy regimens, we were interested in specifying not only which interventions show some scientific evidence of efficacy, but also in application to which kind of chemotherapy regimen.

Of all the interventions analyzed in our review, only three showed significant efficacy in preventing OM (cryotherapy, palifermin and LLLT), while only one intervention proved effective in the treatment of established mu-cositis (LLLT).

Cryotherapy has been one of the most widely investigated interventions over the years. As we have seen, it is very effective in preventing OM in patients scheduled for chemotherapy with antineoplastic drugs that have a short plasma half-life, such as bolus doses of 5-fluorouracil or the administration of high-dose melphalan prior to HSCT.

Another treatment supported by important scientific evidence is palifermin (keratinocyte growth factor). Indeed, it is the only drug approved to date by the United States Food and Drug Administration (FDA) for the prevention of oral mucositis in patients subjected to myeloablative treatment with chemotherapy and radiotherapy prior to HSCT. The clinical trials show the ideal approach in patients of this kind to be the intravenous administration of 60 μg/kg of palifermin a day during three consecutive days before the start of conditioning, with a further three days of administration after stem cell transplantation (days 0, 1 and 2).

In 2011, the studies carried out by Henke et al. (33) and Le et al. (34) indicated that palifermin could also be effective for the prevention of OM in head and neck cancer patients subjected to radiotherapy (total dose 60-66 Gy) together with chemotherapy (cisplatin).

Interest in the development of radiation techniques for the prevention and treatment of OM has grown in recent years. To date, low-level laser therapy (LLLT) is the technique that has shown the greatest effectiveness. Many clinical trials have evaluated the preventive efficacy of LLLT, and the best results have been obtained when it is used in patients subjected to conditioning regimens prior to HSCT.

According to the review published by Clarkson et al. (48), LLLT is the only technique to have shown certain evidence of efficacy in the treatment of established mucositis. This is the conclusion drawn from the clinical trials carried out by Genot-Klastersky et al. (45) and Kuhn et al. (46), in which patients with OM secondary to conditioning prior to HSCT recovered earlier from their oral lesions when laser therapy was applied. Nevertheless, it must be underscored that these are only two studies, and moreover involve few patients (57 in total). The body of evidence for this type of intervention is therefore smaller than in the above cases.

It is also important to emphasize the importance of correct oral hygiene in cancer patients. Although there is no scientific evidence demonstrating the efficacy of oral hygiene protocols in the prevention and treatment of mucositis, the great majority of authors agree that adhesion to oral hygiene measures can reduce the duration and severity of OM (12), as well as help prevent the development of dental problems during the cancer treatment cycles.

The rest of the interventions showed no evidence of benefit, either due to methodological shortcomings of the trials, or because of contradicting results.

Our conclusions coincide with the guidelines the Multinational Association of Supportive Care in Cancer (MASCC) and the International Society of Oral Oncology (ISOO), which were updated in 2012 through several review articles (15,22,29,40).

In sum, at present no concrete intervention may be regarded as the gold standard for the prevention and/or treatment of oral mucositis, and we therefore must resort to recommendations based on the documented evidence. This indicates the need for increasing the methodological quality and number of clinical trials with the aim of expanding the scientific evidence supporting the different interventions. In this context, clinical trials should be carried out following the norms established by the CONSORT (Consolidated Standards of Reporting Trials) declaration, and including sufficient patients and subgroups in accordance to the type of chemotherapeutic agent involved. On the other hand, we consider it very important to unify criteria regarding the use of a single mucositis severity grading system, since this would facilitate comparisons among the different studies. In this respect, we recommend use of the scale developed by the WHO (grades 0-4) ( Table 1).
